# Comparative Microbiota Composition Across Developmental Stages of Natural and Laboratory-Reared *Chironomus circumdatus* Populations From India

**DOI:** 10.3389/fmicb.2021.746830

**Published:** 2021-11-26

**Authors:** Sivan Laviad-Shitrit, Rotem Sela, Yehonatan Sharaby, Leena Thorat, Bimalendu B. Nath, Malka Halpern

**Affiliations:** ^1^Department of Evolutionary and Environmental Biology, University of Haifa, Haifa, Israel; ^2^Department of Zoology, Savitribai Phule Pune University, Pune, India; ^3^Department of Biology, York University, Toronto, ON, Canada; ^4^Department of Biology and Environment, University of Haifa, Haifa, Israel

**Keywords:** microbiota, bacterial composition, chironomids, *Chironomus circumdatus*, developmental stages, *Cetobacterium*

## Abstract

Chironomids are aquatic insects that undergo a complete metamorphosis of four life stages. Here we studied, for the first time, the microbiota composition of *Chironomus circumdatus*, a tropical midge species, both from the Mula and Mutha Rivers in Pune, India and as a laboratory-reared culture. We generated a comparative microbial profile of the eggs, larvae and pupae, the three aquatic life stages of *C. circumdatus*. Non-metric multidimensional scaling analysis (NMDS) demonstrated that the developmental stage had a more prominent effect on the microbiota composition compared to the sampling location. Notably, the microbiota composition of the egg masses from the different sampling points clustered together and differed from laboratory culture larvae. *Proteobacteria* was the dominant phylum in all the environmental and laboratory-reared egg masses and pupal samples, and in the laboratory-reared larvae, while *Fusobacteria* was the dominant phylum in the larvae collected from the field environment. The most abundant genera were *Cetobacterium*, *Aeromonas*, *Dysgonomonas, Vibrio*, and *Flavobacterium*. The ten amplicon sequence variants (ASVs) that most significantly contributed to differences in microbiota composition between the three sampled locations were: *Burkholderiaceae* (ASVs 04 and 37), C39 (*Rhodocyclaceae*, ASV 14), *Vibrio* (ASV 07), *Arcobacter* (ASV 21), *Sphaerotilus* (ASV 22), *Bacteroidia* (ASVs 12 and 28), *Flavobacterium* (ASV 29), and *Gottschalkia* (ASV 10). No significant differences were found in the microbial richness (Chao1) or diversity (Shannon H’) of the three sampled locations. In contrast, significant differences were found between the microbial richness of the three life stages. Studying the microbiota of this *Chironomus* species may contribute to a better understanding of the association of *C. circumdatus* and its microbial inhabitants.

## Introduction

Chironomids (*Diptera: Chironomidae*), also known as non-biting midges, are aquatic insects that undergo a complete metamorphosis of four life stages (egg, larva, pupa, and adult). Females of the genus *Chironomus* deposit egg masses on biotic or abiotic objects that float on the surface of water bodies. A single egg mass contains hundreds of eggs that are embedded in a glycoprotein and chitin gelatinous matrix (Halpern et al., 2003; Laviad et al., 2016). The first larval instars feed on the egg mass remains and then swim toward the bottom of the water body where they build a silken tube (Thorat and Nath, 2018). The larval foraging zone is limited to the tube entrance (Halpern et al., 2002). In some species, the larvae feed by extending their head and anterior part of their body outside the tube. These housing-cum-feeding tubes protect the larvae from their fish and invertebrate predators and other environmental stressors (Macchiusi and Baker, 1992; Thorat et al., 2017, 2020; Laviad-Shitrit et al., 2021). The fourth instars metamorphose into pupae and remain within the tube at the bottom of the water habitat. Next, the pupa inflates using a gas bubble that separates the pupal skin from the pharate adult, reaches the water surface, and emerges into an adult within 10–30 s (Oliver, 1971; Brackenbury, 2000). Adults are non-aquatic and males create aerial mating swarms. After fertilization, the females deposit egg masses in water.

Chironomids inhabit almost all aquatic habitats and usually are the dominant insect in their habitats (Halpern and Senderovich, 2015). Chironomids are critical components in the aquatic food chain due to their contribution to the sustenance of the aquatic ecosystem (Hölker et al., 2015). In one site there can be up to 100 different chironomid species (Ferrington, 2008). The differences in the presence and abundance of chironomid species in lakes can occur due to different environmental conditions including water depth (Pleskot et al., 2019).

Insects are associated with a variety of microorganisms that can be either vertically transmitted or acquired from the environment (Gurung et al., 2019). Microbiota in insects have been found to change between life stages and can influence different aspects of insect development, physiology, and ecology (Feldhaar, 2011; Engel and Moran, 2013; Sela et al., 2020). Hammer and Moran (2019) found that the insect-microbiota association changes across metamorphosis and is different even between related insect species. Endogenous microbiota contributes to an insect host’s dietary supplementation, helps in coping with temperature stress and toxic substances, and abiotic protection and enhances the host immune system (Senderovich and Halpern, 2013; Gurung et al., 2019).

Rouf and Rigney (1993) were the first to study the bacterial composition of chironomid larvae using culturable methods. Following their work, a few other studies were conducted on the bacterial composition of chironomid egg masses focusing on the presence of *V. cholerae* and *Aeromonas* species (Halpern et al., 2004, 2006, 2007; Senderovich et al., 2008; Senderovich and Halpern, 2012, 2013; Laviad et al., 2016; Sela and Halpern, 2019), and on the presence of *V. cholerae* in chironomid larvae and exuviae (Senderovich and Halpern, 2012, 2013; Lotfi et al., 2016; Sela et al., 2020). Sela et al. (2020) compared the bacterial composition of *Chironomus transvaalensis* egg masses, larvae, pupae, and adults. Recently, Laviad-Shitrit et al. (2020) sampled nine chironomid species from the Mula and Mutha Rivers in India and from a waste stabilization pond in Israel and found evidence for the presence of toxigenic O1 and O139 *V. cholerae* serogroups in these chironomid species.

Here we studied the microbiota composition of *Chironomus circumdatus* egg masses, larvae, and pupae that were sampled from the Mula and Mutha Rivers in Pune, India, and from a laboratory culture. To the best of our knowledge, this is the first study that characterizes the bacterial composition of *C. circumdatus* and also the first in which the microbiota composition of any *Chironomus* species is compared between different environmental habitats. Significant differences were found between the microbiota compositions of the different developmental stages of *C. circumdatus* but not between the microbiota compositions of the same life stage sampled from different habitats.

## Materials and Methods

### *Chironomus circumdatus* Sampling

*C. circumdatus* egg masses, larvae, and pupae were collected in November 2018 from plants (*Eichhornia crassipes*) and riverbank sediments from urban settings surrounding two rivers in Pune, India: (i) the Mula River (18.5551° N, 73.8618° E) and (ii) the Mutha River (18.2901° N, 73.4956° E) (Laviad-Shitrit et al., 2020). Samples were also collected from (iii) laboratory cultures being reared in the Animal House Facility at the Department of Zoology, SPPU, Pune, India.

For the laboratory culture, fourth larval instars were picked from sediments at the Mula riverbank 8 months prior to the sampling for the current study. Larvae collected from the field were reared in the laboratory, and egg masses, larvae, and pupae from subsequent generations were used in this study as laboratory samples (hereafter, laboratory culture). Cultures were maintained in non-toxic plastic tubs (diameter = 35 cm) in an animal house facility at the Department of Zoology (SPPU, India) following ethical guidelines. Culture tubs were layered with sterilized beach sand as an inert substratum material at the base and were provided with fresh water and food every alternate day. Tap water was boiled and filtered to make it potable and microbe-free before feeding the animals. Food mixture comprised of *Sphagnum* moss and baker’s yeast (5:1). The moss was washed in sterile water, soaked overnight in hot water (70–80°C) and thoroughly rinsed the following day before grinding it to prepare the food diet. Larval transition to the pupal stage was monitored and mature pupae were collected and maintained in culture tubs inside cages. Adults that emerged were allowed to mate and egg ropes laid on the water surface were carefully collected into fresh tubs to raise the progeny.

All the samples that were investigated in the current study were taxonomically identified as *C. circumdatus* by Laviad-Shitrit et al. (2020). Each sample was treated individually after collection by washing it five times in 1 ml sterile water to remove the microorganisms that were not tightly connected to the samples. All samples were then preserved in storage tubes containing absolute ethanol at –20°C until DNA extraction. Overall, 102 *C. circumdatus* specimens were collected. The details regarding the number of the specimens and their life stage and sampling location are summarized in Supplementary Table 1.

### DNA Extraction

To remove ethanol residues, samples were centrifuged for 30 min at maximum speed, and the ethanol was removed using a sterile pipette tip. Each individual sample was then incubated in a heat block for 10 min at 100°C, washed in sterile saline water, and crushed with a sterile homogenizer.

DNA was extracted from each sample using a DNA isolation kit (DNeasy Blood and Tissue, Qiagene, Germany) according to the manufacturer’s instructions with minor modifications, as was described previously (Laviad-Shitrit et al., 2017). DNA samples were stored at –20°C.

### *C. circumdatus* Microbiota Identification Using 16S rRNA Illumina Sequencing

Genomic DNA was PCR amplified with primers targeting the V4 region of the bacterial 16S rRNA gene. The primers were modified from the primer set employed by the Earth Microbiome Project; CS1_515F (ACACTGACGACATGGTTCTACAGTGCCAGCMGCCGCGG TAA) and CS2_806R (TACGGTAGCAGAGACTTGGTCTGGA CTACHVGGGTWTCTAAT) (Caporaso et al., 2012) (Sigma Aldrich, Israel). The primers contained 5′ common sequence tags (known also as common sequence 1 and 2, CS1 and CS2). Amplicons were created using two-stage “targeted amplicon sequencing (TAS)” as described previously (Naqib et al., 2018).

PCR was performed as described previously (Sela et al., 2020). Sterile DNA-free water was used as a negative control for DNA extraction and PCR amplification to verify that there was no contamination. No contamination was found.

Additional PCR amplification was performed in 10 μl reactions in 96-well plates using MyTaq HS 2X master mix (Bioline, London, United Kingdom). Each sample had a separate primer pair that contained a unique 10-base barcode obtained from the Access Array Barcode Library for Illumina (Fluidigm, South San Francisco, CA, United States; Item# 100–4876). These Access Array primers contained the CS1 and CS2 linkers at the 3′ ends of the oligonucleotides. The conditions for the second PCR and the procedure of the Illumina sequencing were as described in Sela et al. (2020). The second PCR amplification and Illumina MiniSeq sequencing were performed at the Genome Research Core (GRC) at the University of Illinois at Chicago (UIC). Sequence length in the Illumina MiniSeq mid-output flow cell was 2 × 150 paired-end reads.

### Sequence Analysis

Overall, 408 fastq files were obtained, corresponding to 102 samples (four files for each sample), with two pair-end sequences each. Data quality of the samples was tested with the fastQC^1^ program which is a quality control tool for high throughput sequence data. All the samples were found to be of high quality.

Data pre-processing was performed using the DADA2 pipeline (DADA2 package version 1.14.0, Callahan et al., 2016). A detailed description of the pre-processing steps is given in Laviad-Shitrit et al. (2021). Following data cleaning, both runs were merged by sample, and checked for chimeras. Suspected chimeras were detected and removed using the command “removeBimeraDenovo.” Then a count table for each amplicon sequence variant (ASV) in each sample was produced. Taxonomic assignment of the ASVs was done using DADA2 assignTaxonomy command with the Silva rRNA database (version 138) as reference and minimum bootstrap value of 80%. Then, ASVs of non-bacterial origin (Archaea, chloroplasts, and mitochondria, as well as unclassified taxa at the phylum level), were filtered out. In addition, all ASVs with sequence lengths below 260 bp or above 262 bp were removed. Following this filtering step, sequences were binned into 3,366 bacterial ASVs and then, ASVs with < 50 reads across the dataset were removed from all analyses. 1,959 ASVs were left after this filtration step. The sequencing coverage per sample (read number) after each of the filtration steps is presented in Supplementary Table 2. The distribution of the abundance of each ASV can be found in Supplementary Table 3. Supplementary Table 3 demonstrates the justification for the deletion of ASVs with < 50 reads across the dataset. The final feature table of the ASVs’ taxonomic classification and abundances within each sampling group after the filtration steps (ASVs > 50) is presented in Supplementary Table 4.

Raw reads were recovered as fastq files and are available in the NCBI database under BioProject accession number PRJNA678840.

### Statistical Analysis

Rarefaction curves were calculated for each sample using Past 4^2^. The curves demonstrate that the sequencing effort was sufficient to effectively represent the bacterial community composition (Supplementary Figure 1). To determine the effect of sampling location and chironomid developmental stage on microbiota composition, Permutation Analysis of Variance (999 permutations) was conducted using the R software (R Core Team, 2018) vegan package (Oksanen et al., 2007). The model was designed with the developmental stage nested within the sampling location and the bacterial community dissimilarity matrix was calculated using the Bray-Curtis index. Non-metric multidimensional scaling (NMDS) based on Bray-Curtis distances was calculated using PRIMER v.7 (Clarke and Gorley, 2015). NMDS visualizes the differences in the bacterial communities’ composition between the different life stages and sampling locations.

Venn diagrams that represent the distinctive and shared ASVs between each of the developmental stages and locations were created by InteractiVenn software^3^ (Heberle et al., 2015).

Bacterial richness (Chao1) and diversity (Shannon H’) were calculated using the alpha-diversity analysis tool in the MicrobiomeAnalyst website^4^ (Dhariwal et al., 2017) and visualized with Microsoft excel 2019. These parameters were compared by non-parametric tests (Kruskal-Wallis), followed by *post hoc* tests using two-sided Mann–Whitney tests adjusted for multiple comparisons with Bonferroni correction (IBM SPSS v.25.0.0.1). The results are presented as mean ± standard error of the mean (SEM).

Linear discriminant analysis Effect Size (LEfSe) was also performed using the microbiome analyst tool (Dhariwal et al., 2017). The analysis was applied on rarefied count data in order to identify the bacterial ASVs that contributed to differences in microbiota composition between the three sampled locations.

## Results

### Microbiota Composition of the Different Life Stages vs. Sampling Locations

The microbiota compositions of the three life stages of *C. circumdatus* that were sampled from three environments were studied using Illumina sequencing of the 16S rRNA gene.

**FIGURE 1:**
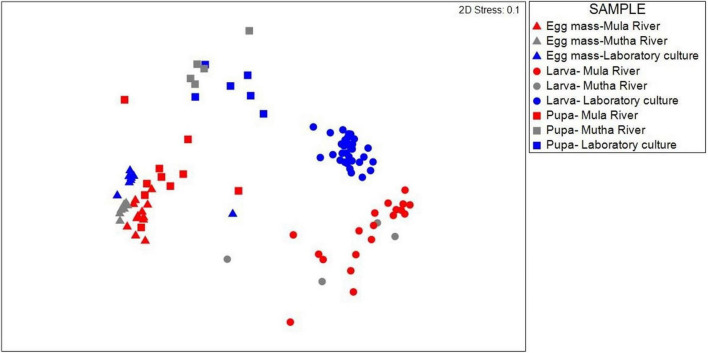
NMDS (non-metric multidimensional scaling) plot based on Bray-Curtis similarity of *C. circumdatus* life stages that were sampled from Mula and Mutha Rivers and from the laboratory culture in Pune, India (stress value = 0.1, *n* = 102). Permutation analysis of variances revealed significant effects of both the chironomid developmental stage and the sampling location on the bacterial community composition (*p* < 0.001, Table 1). Although significant differences were found between the life stages from the different locations, the bacterial communities of egg masses from the different sampling locations clustered together.

Similarities between the life stages from the different sampling locations were examined using NMDS (Figure 1). Permutation analysis of variances revealed significant effects of both the chironomid developmental stage and the sampling location on the bacterial community composition (*p* < 0.001, Table 1). The model attributed approximately 54% of the variation in bacterial community compositions to these two factors. The developmental stage had a more prominent effect [*F*_(6, 93)_ = 13.76, *R*^2^ = 0.41, *p* = 0.001] compared to the sampling location [*F*_(2, 93)_ = 13.23, *R*^2^ = 0.13, *p* = 0.001]. Nevertheless, the bacterial communities of egg masses from the different sampling points clustered together (Figure 1). In addition, the environmental larval samples either clustered together or differed from the bacterial community composition of the laboratory larval culture.

**TABLE 1:** Permutation analysis of variances results testing the significant effects of both the chironomid developmental stage and the sampling location on the bacterial community composition.

	Df	Sums of Sqs	Mean Sqs	F. Model	*R* ^2^	*p*-value
Location	2	4.92	2.46	13.23	0.13	0.001
Stages	6	15.36	2.56	13.75	0.41	0.001
Residuals	93	17.31	0.19		0.46	
Total	101	37.59			1.00	

*The model attributed approximately 54% of the variation in bacterial community compositions to these two factors. The developmental stage had a more prominent effect compared to the sampling location.*

### Bacterial Richness and Diversity

**FIGURE 2:**
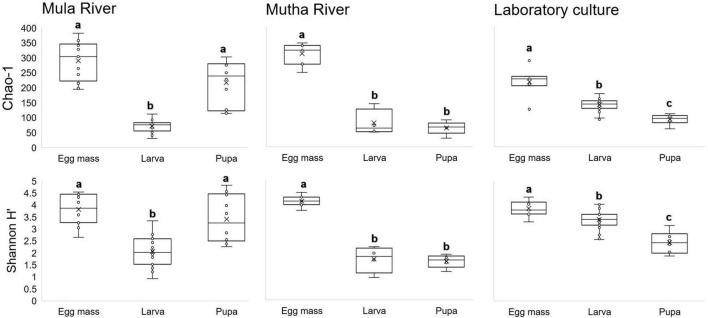
Box-plots depicting bacterial ASV richness and Shannon H’ diversity, for the different metamorphosis life stages’ bacterial community compositions in the samples from three locations: Mula and Mutha Rivers and a laboratory culture. No significant differences were found for the microbial richness (Chao1) and the bacterial diversity (Shannon H’) among the three sampling locations (Kruskal-Wallis: H_2_ = 0.13, *p* = 0.94; H_2_ = 4.45, *p* = 0.108, respectively). Significant differences were found between the microbial richness (Chao1) of the three life stages (Kruskal-Wallis: H_2_ = 46.99, *p* < 0.0001). Richness was the highest in the egg mass stage. Different letters represent significant differences with a 95% confidence interval.

No significant differences were found in microbial richness (Chao1) for the three sampling locations (Mula River, Mutha River, and laboratory culture) (Kruskal-Wallis: H_2_ = 0.13, *p* = 0.94). Similar results were obtained when the bacterial diversity (Shannon H’) was compared for these three sampling locations as well (Kruskal-Wallis: H_2_ = 4.45, *p* = 0.11). However, significant differences were found between the microbial richness (Chao1) of the three life stages (Kruskal-Wallis: H_2_ = 46.99, *p* < 0.001). Richness was the highest in the egg masses stage (2.2, and 1.95 times greater compared to the richness of the larval and pupal stages, respectively). Richness values were significantly different between the egg masses and the larvae, and the egg masses and the pupae (Mann–Whitney: U = 29, *p* < 0.001; U = 71, *p* < 0.001, respectively) (Figure 2).

When bacterial diversity (Shannon H’) was compared between the three developmental stages (egg masses, larvae, and pupae), significant differences were observed (Kruskal-Wallis: H_2_ = 330.46, *p* < 0.001) most notably between the egg masses and the larvae (Mann–Whitney: U = 176, *p* < 0.001) and the egg masses and the pupae (Mann–Whitney: U = 96, *p* = 0.001). Shannon diversity was the highest in the egg masses (1.8, and 2 times greater than the diversity of the larval, and pupal stages, respectively) (Figure 2).

### Microbial Taxonomy

A total of 1,959 ASVs were detected and of those, 1,026 ASVs were classified into 306 known bacterial genera. The other 933 ASVs (47.62% of the total ASVs) did not have a taxonomic identification at the genus-level. The average prevalence of the genus-level unclassified ASVs was 37.58% out of all the sequences. *Aeromonas* was the most prevalent genus, detected in 100 samples, with a mean relative abundance of 4.77 ± 1.18%. The most abundant genus was *Cetobacterium* with 14.20 ± 2.03% relative abundance; however, it was less prevalent than *Aeromonas*, and it was identified in only 88 samples. Other abundant genera were *Dysgonomonas* (3.69 ± 0.61%), *Vibrio* (3.49 ± 1.1%), and *Flavobacterium* (2.39 ± 0.34%).

Overall, 31 bacterial phyla were detected across the entire dataset. *Proteobacteria, Bacteroidetes*, and *Firmicutes* were the most prevalent (detected in all 102 samples) followed by *Fusobacteria* (98 samples), *Epsilonbacteraeota*, *Actinobacteria* (96 samples each), and *Cyanobacteria* (90 samples). *Proteobacteria* was also the most abundant phylum with 45.39 ± 2.49% of the ASVs followed by *Bacteroidetes* (18.81 ± 1.12%), *Fusobacteria* (14.54 ± 2.01%), and *Firmicutes* (10.35 ± 1.12%).

**FIGURE 3:**
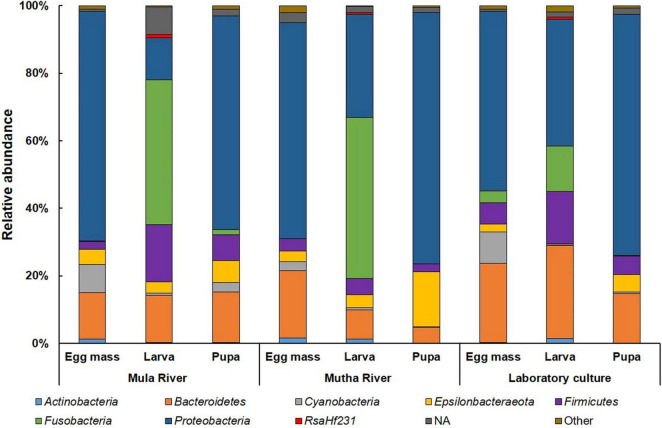
Average ASV relative abundances at the phylum level. All the life stages for each sampling location are presented. *Proteobacteria* was the dominant phylum in the environmental and laboratory egg masses and pupal samples and in the laboratory larvae. *Fusobacteria* was the most dominant phylum in the environmental larvae. More details can be found in Supplementary Table 5.

*Proteobacteria* was the most dominant phylum in all the environmental and laboratory egg masses and pupal samples and in the laboratory-reared larvae (Figure 3 and Supplementary Table 5), while *Fusobacteria* was the most dominant phylum in the environmental larvae (Figure 3). *Firmicutes* was found in relatively high abundance in the larval samples from Mula River and from the laboratory (17.00 ± 3.12%, and 15.42 ± 1.58% respectively). ASVs belonging to the phylum *Epsilonbacteraeota* were relatively abundant in the pupal samples from Mutha river (16.40 ± 2.97%) while ASVs belonging to the phylum *Cyanobacteria* were abundant in the egg masses from Mula River and from the laboratory culture (8.30 ± 3.44%, and 9.46 ± 2.01%) (Figure 3 and Supplementary Table 5).

The genera *Flavobacterium* and *Planktothricoides* SR001 were found in high abundance in all the egg masses from the three sampling locations (4.71–10.97% and 1.79–8.04% of the ASVs, respectively) (Table 2). *Cetobacterium* was found in high abundance in all the larval samples from the three sampling locations (between 13.52 and 47.62% of the ASVs). *Dysgonomonas* was found in high abundance larvae that were sampled from in Mula River and the laboratory culture (5.84 ± 1.24 and 5.64 ± 0.58%, respectively) (Table 2). *Arcobacter* and *Aeromonas* were found in high abundance in all pupal samples from all the three sampling points (5.05–16.18% and 9.33–17.73% of the ASVs, respectively). The genus *Vibrio* was found in high abundance in the egg masses, larvae, and pupae that were sampled from the Mula River (13.24 ± 4.28, 6.12 ± 4.18, and 6.89 ± 4.64%, respectively) (Table 2).

**TABLE 2:** Taxa with relative abundance (%) of at least 5.0% in at least one of the samples.

Class	Genus	Mula River			Mutha River			Laboratory culture		
		Egg mass	Larva	Pupa	Egg mass	Larva	Pupa	Egg mass	Larva	Pupa
*Bacteroidia*	*Dysgonomonas*	0.00 ± 0.00	**5.84 ± 1.24**	4.82 ± 4.68	0.00 ± 0.00	3.23 ± 1.95	0.64 ± 0.62	2.62 ± 2.62	**5.64 ± 0.58**	0.34 ± 0.24
*Bacteroidia*	*Flavobacterium*	**5.28 ± 0.84**	0.16 ± 0.05	1.61 ± 0.46	**10.97 ± 1.50**	0.20 ± 0.07	2.48 ± 0.98	4.71 ± 0.86	0.46 ± 0.05	3.96 ± 1.22
*Bacteroidia*	*Paludibacter*	0.45 ± 0.08	0.00 ± 0.00	0.10 ± 0.04	0.51 ± 0.23	0.01 ± 0.01	0.001 ± 0.001	**6.77 ± 1.47**	0.002 ± 0.001	0.01 ± 0.007
*Campylobact eria*	*Arcobacter*	3.42 ± 0.72	0.12 ± 0.005	**5.67 ± 2.88**	1.77 ± 0.33	2.60 ± 2.53	**16.18 ± 3.28**	0.75 ± 0.29	0.01 ± 0.004	**5.05 ± 3.59**
*Clostridia*	*Gottschalkia*	0.00 ± 0.00	**6.58 ± 1.56**	0.07 ± 0.05	0.00 ± 0.00	0.55 ± 0.52	0.08 ± 0.08	1.14 ± 1.13	2.85 ± 0.87	0.03 ± 0.02
*Fusobacteriia*	*Cetobacterium*	0.04 ± 0.01	**42.92 ± 4.23**	0.39 ± 0.25	0.16 ± 0.006	**47.61 ± 11.87**	0.009 ± 0.005	0.69 ± 0.67	**13.52 ± 2.30**	0.12 ± 0.09
*Gammaproteo bacteria*	*Acidovorax*	2.88 ± 0.48	0.01 ± 0.01	0.74 ± 0.16	1.86 ± 0.25	0.03 ± 0.03	0.04 ± 0.02	**5.61 ± 1.73**	0.01 ± 0.005	0.04 ± 0.02
*Gammaproteo bacteria*	*Acinetobacter*	**5.58 ± 1.44**	0.25 ± 0.15	1.26 ± 0.55	1.21 ± 0.55	0.44 ± 0.40	0.02 ± 0.006	0.27 ± 0.14	0.10 ± 0.03	0.06 ± 0.02
*Gammaproteo bacteria*	*Aeromonas*	1.43 ± 0.34	0.39 ± 0.17	**17.73 ± 6.17**	0.67 ± 0.30	1.56 ± 0.86	**17.26 ± 14.80**	1.61 ± 0.57	3.76 ± 1.41	**9.33 ± 6.12**
*Gammaproteo bacteria*	C39*	**6.10 ± 4.62**	0.05 ± 0.02	0.25 ± 0.08	**21.58 ± 2.14**	0.002 ± 0.002	0.10 ± 0.09	0.34 ± 0.07	0.001 ± 0.001	0.005 ± 0.005
*Gammaproteo bacteria*	*Sphaerotilus*	0.02 ± 0.007	0.015 ± 0.012	0.04 ± 0.02	0.03 ± 0.01	0.02 ± 0.01	**12.80 ± 2.62**	0.006 ± 0.006	0.09 ± 0.08	**12.26 ± 3.73**
*Gammaproteo bacteria*	*Vibrio*	**13.24 ± 4.28**	**6.12 ± 4.18**	**6.89 ± 4.64**	0.84 ± 0.19	0.01 ± 0.009	0.00 ± 0.00	0.49 ± 0.47	0.06 ± 0.03	0.04 ± 0.03
*Oxyphotobact eria*	*Planktothricoi des* SR001*	**6.31 ± 3.12**	0.06 ± 0.04	0.24 ± 0.10	1.79 ± 0.60	0.00 ± 0.00	0.02 ± 0.02	**8.04 ± 1.99**	0.004 ± 0.002	0.004 ± 0.003

*The results are presented as mean ± standard error of the mean (SEM). Genera with abundances over 5.0% are marked in bold.*

*unidentified genera (n = 102).

### Common and Specific Amplicon Sequence Variants Analysis

**FIGURE 4:**
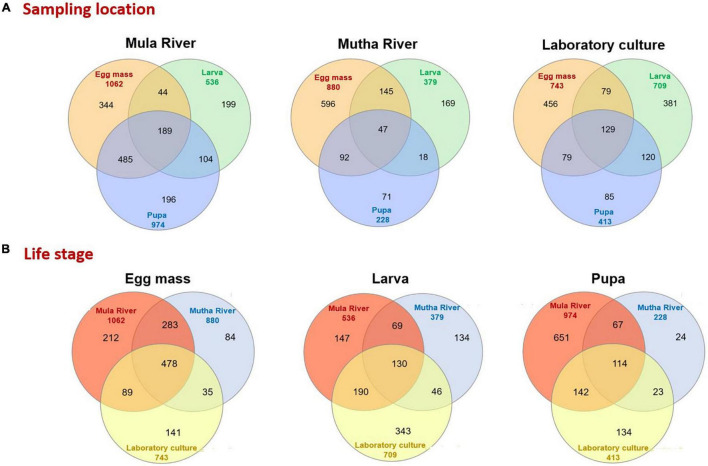
Venn diagram representing the number of shared and unique ASVs in the bacterial communities of the different life stages that were sampled from the different environments. **(A)** Sampling location. A Venn diagram of the developmental life stages microbiota for each sampling location. **(B)** Life stage. A Venn diagram that compares the sampling locations for each life stage (egg mass, larva, pupa).

The distinctive and shared ASVs of the different developmental stages from the different sampling points were analyzed using Venn diagrams (Figure 4). Only a minority of the ASVs were shared between the three life stages in the different sampling locations (Mula River, Mutha River, and laboratory culture: 12.1, 4.1, and 9.7%, respectively, Figure 4A). The highest percentages of shared ASVs were observed in the egg masses from the three sampling points (32.4–67.7% unique ASVs, Figure 4A). Egg masses and pupae from the Mula River shared the highest portion of ASVs (33.4%). In contrast, in the Mutha River, egg masses and larvae shared the highest portion of ASVs (15.2%, Figure 4A). Comparisons of the shared and the unique ASVs of each life stage between the different sampling points showed that life stages from the different sampling points harbored a small portion of shared ASVs (Figure 4B). For each life stage, only a minority of the ASVs were shared across the three sampling locations (egg masses, larvae, and pupae: 17.8, 8.0, and 7.0%, respectively) (Figure 4B).

### Linear Discriminant Analysis

To identify which ASVs contributed significantly to the variation in the microbiota of the different sampling locations, we used LDA effect size (LEfSe) (Supplementary Figure 2 and Supplementary Table 6). The ten ASVs that most significantly contributed to differences in microbiota composition between the three sampled locations were: *Burkholderiaceae* (ASV 04), C39 (*Rhodocyclaceae*, ASV 14), *Vibrio* (ASV 07), *Burkholderiaceae* (ASV 37), *Arcobacter* (ASV 21), *Sphaerotilus* (ASV 22), *Bacteroidia* (ASV 12), *Flavobacterium* (ASV 29), *Gottschalkia* (ASV 10), and *Bacteroidia* (ASV 28). In the Mula River, *Vibrio*, and *Gottschalkia* were the genera with the highest abundances of all discriminant ASVs. In the Mutha River, unclassified *Burkholderiaceae*, C39, *Arcobacter, Sphaerotilus*, and *Flavobacterium* were the most abundant of all discriminant ASVs. In the laboratory samples, the two unclassified *Bacteroidia* ASVs 12 and 28, were the most abundant (Supplementary Figure 2 and Supplementary Table 6).

## Discussion

*C. circumdatus* is one of the most common and wide-spread chironomid species in India (Kumar and Gupta, 1990). To the best of our knowledge, this is the first study that investigates the changes in the bacterial composition at the different metamorphic life stages of *C. circumdatus* and compares them across different sampling locations. Our results demonstrate noticeable compositional differences, more so in a developmental stage-specific manner rather than in a sampling location-specific manner. The larval microbiota that was sampled from the rivers was similar and differed from the larval microbiota composition from the laboratory culture. Chironomid larvae are known as opportunistic omnivores that ingest a wide variety of food items (Cummins and Klug, 1979). Chironomid larvae feed on algae, fungi, pollen, leaves, and wood remains as well as animal remains, detritus, and silt (Henriques-Oliveira et al., 2003). The differences in the diets between the free-living and the laboratory sampled larvae are likely the driving factor that resulted in differences between the larval bacterial composition of larvae that were sampled from the rivers and the laboratory (Figure 1).

Similar to what was found in the current study, Sela et al. (2020), who studied the bacterial composition of *C. transvaalensis* life stages, found significant differences between the microbiota compositions of the different developmental stages. Developmental stage was also found to be a factor that affected the bacterial composition in mosquito species such as *Aedes koreicus*, *Anopheles coluzzii*, *Anopheles gambiae*, and *Culex tarsalis* (Wang et al., 2011; Gimonneau et al., 2014; Duguma et al., 2015; Bascuñán et al., 2018; Alfano et al., 2019). Similarly, significant differences were also found between the bacterial composition of different life stages of the butterfly *Heliconius erato* (Hammer and Moran, 2019) and the moth *Brithys crini* (González-Serrano et al., 2019).

Gimonneau et al. (2014) found that the breeding site of the larvae is a crucial factor that shapes the community composition in adults. Moreover, Bascuñán et al. (2018) found that mosquito developmental stage and the geographical location are more important than species or adult feeding status in determining gut bacterial composition in *Anopheles nuneztovari* and *Anopheles darling*. However, no such pattern was found in the microbiota composition of the metamorphic developmental life stages of leaf-cutting ants *Acromyrmex echinatior* and *Atta cephalotes* (Zhukova et al., 2017).

The bacterial richness and diversity (Chao1 and Shannon H’ indices) showed no significant differences in the bacterial compositions of the life sages that were sampled from the three sampling points. However, richness differed significantly between the microbiota composition of the different life stages. The richness of the egg masses was the highest, followed by the larval and pupal microbiota richness in the Mutha River and the laboratory culture (Figure 2). The larval microbiota composition in culicine mosquitoes and in the butterfly *H. erato* also showed high richness and diversity compared to the pupae (no egg masses were sampled) (Hammer et al., 2014; Duguma et al., 2015).

A decline in bacterial diversity at the ASVs level was observed in the samples from the three locations as the insect developed from egg masses to pupae. Similar results were obtained when the microbial composition of different metamorphic life stages of *C. transvaalensis* were studied (Sela et al., 2020). A decrease in the bacterial diversity between the larval and the adult stages was also observed in the moth *B. crini*. The authors suggested that the decline in the bacterial diversity toward the adult stage was a result of changes in adult intestine structure (González-Serrano et al., 2019).

Venn diagram showed that egg masses from all the sampling points harbor the highest percent of unique ASVs. These results do not match with previous findings for *C. transvaalensis* (Sela et al., 2020).

*Proteobacteria* was the dominant phylum in all the environmental and laboratory egg masses and pupal samples, while *Fusobacteria* was the dominant phylum in the environmental larvae. Similarly, when microbial compositions of the different *C. transvaalensis* life stages were studied, *Proteobacteria* was the most dominant phylum, while *Fusobacteria* showed relatively high abundance in the larval microbiome (Sela et al., 2020).

The most abundant genera in the current study were *Cetobacterium*, *Aeromonas*, *Dysgonomonas, Vibrio*, and *Flavobacterium*. *Cetobacterium* comprised almost half (43–48%) of the bacterial community in the larvae that were sampled from the Mula and Mutha Rivers. Their relative abundance decreased in the laboratory larval microbiota to about 14% of the microbiota composition. This implies that *Cetobacterium* has an important role in the larval life stage. The high abundance of this genus in the larvae is more pronounced when it is compared to its abundance in the egg mass and pupa life stages (nearly absent, in all habitats) (Table 2). Similarly, *Cetobacterium* was highly abundant in *C. ramosus* larval microbiota that were sampled from the Mutha River (39.9%) while its abundance in laboratory culture was reduced to 3.3% (Sela et al., 2021). This suggests that *Cetobacterium* has a role in the larval survival in the river. Interestingly, *Cetobacterium* was not identified in any of *C. transvaalensis* life stages that were sampled from waste stabilization ponds in Israel (Senderovich and Halpern, 2013; Sela et al., 2020). *Cetobacterium* is comprised of two described species *C. ceti* and *C. somerae*. These species are found in high prevalence in the gastrointestinal tracts of different fish species (LaFrentz et al., 2020). Fish feed on chironomid larvae, and this may explain the presence of *Cetobacterium* in the fish gut (Halpern and Izhaki, 2017). The contribution of *Cetobacterium* to the chironomid larval life stage still stands to be explored. *Cetobacterium somerae* is an acetate producer and it was recently found that increased abundance of acetate-producing *Cetobacterium* contributed to glucose homeostasis in fish (Wang et al., 2021). According to Wang et al. (2021), *Cetobacterium* improves fish carbohydrate utilization. Sugita et al. (1991) found that *Cetobacterium* promotes the synthesis of vitamin B12.

*Flavobacterium* was highly abundant in the egg masses that were sampled from the rivers. This genus is widely distributed in aquatic ecosystems (streams, rivers, lakes, muddy soils, wastewater, groundwater, ponds, etc.) and some species are the etiological agents of fish diseases (Bernardet and Bowman, 2006). *Dysgonomonas* was detected in high prevalence in the larvae that were sampled in the Mula River and the laboratory culture. Recently it was found that when *C. transvaalensis* larvae were exposed to toxic hexavalent chromium, the relative abundance of *Dysgonomonas* increased in the larval microbiome compared to control larvae (Laviad-Shitrit et al., 2021). The genus was detected previously in the mosquito and fruit fly and has been associated with symbiosis, host immunology, and developmental biology (Bridges and Gage, 2021).

The dominant genera that were detected in relatively high prevalence in all the pupal samples were *Arcobacter* and *Aeromonas* (Table 2). *Arcobacter* is known as a serious hazard to human health and a significant zoonotic pathogen (Collado and Figueras, 2011). *Aeromonas* is an important disease-causing pathogen of fish and other cold-blooded species and is also pathogenic to humans (Janda and Abbott, 2010). *Vibrio* was detected at high prevalence in all the chironomid life stages from the Mula River and was one of the genera with the highest abundances of all discriminant ASVs (Supplementary Figure 2 and Supplementary Table 6). *Vibrio*, ubiquitous in the Mula River samples (Table 2), was significantly reduced in the laboratory-reared culture, demonstrating that *Vibrio* may contribute to the insects’ survival in the river. *V. cholerae* was described previously as part of the bacterial composition of chironomids (Broza and Halpern, 2001; Halpern and Senderovich, 2015; Laviad and Halpern, 2016). Recently, it was found that different chironomid species, including *C. circumdatus*, harbor toxigenic *V. cholerae* strains (O1 and O139) (Laviad-Shitrit et al., 2020). In the Mutha River, unclassified *Burkholderiaceae* (ASVs 04 and 37), C39 (*Rhodocyclaceae*), *Arcobacter, Sphaerotilus*, and *Flavobacterium* were the most abundant of all discriminant ASVs (Supplementary Figure 2 and Supplementary Table 6). The role of these genera in the insects’ survival in the environment has yet to be explored.

To conclude, specific genera were most dominant in specific life stages of *C. circumdatus* metamorphosis. *Flavobacterium* and C39 (*Rhodocyclaceae*) were the most dominant genera in egg masses that were sampled from the environment, *Cetobacterium* constituted most of the larval microbiota composition (almost 50%) and *Arcobacter* and *Aeromonas* were the dominant genera in the pupal life stage (Table 2). This dominance was found in the life stages that were sampled from all the three sampling sites, demonstrating that these genera are stable residents in each of *C. circumdatus* life stages and the sampling location’s influence on the microbiota composition is minor. Recently, the presence of toxigenic *Vibrio cholerae* serogroup O1 and the cholera toxin genes were detected in different life stages of *C. circumdatus* (Laviad-Shitrit et al., 2020). Thus, integrated and multidisciplinary studies on microbiota composition will provide a deeper understanding of the association of *V. cholerae* and other microbial inhabitants with *C. circumdatus.*

## Data Availability Statement

The datasets presented in this study can be found in online repositories. The names of the repository/repositories and accession number(s) can be found below: https://www.ncbi.nlm.nih.gov/, PRJNA678840.

## Ethics Statement

Ethical review and approval was not required for the animal study because the study was conducted on chironomids (Insects).

## Author Contributions

SL-S, LT, BBN, and MH conceived of and designed the experiments. SL-S performed the experiments and wrote the manuscript. SL-S, YS, RS, and MH analyzed the data. BBN and MH contributed reagents, materials, and analysis tools. RS, YS, LT, BBN, and MH reviewed and commented on the manuscript. All authors contributed to the article and approved the submitted version.

## Conflict of Interest

The authors declare that the research was conducted in the absence of any commercial or financial relationships that could be construed as a potential conflict of interest.

## Publisher’s Note

All claims expressed in this article are solely those of the authors and do not necessarily represent those of their affiliated organizations, or those of the publisher, the editors and the reviewers. Any product that may be evaluated in this article, or claim that may be made by its manufacturer, is not guaranteed or endorsed by the publisher.

## Footnotes

1
https://www.bioinformatics.babraham.ac.uk/projects/fastqc

2
https://www.nhm.uio.no/english/research/infrastructure/past/

3
http://www.interactivenn.net/index.html

4
https://www.microbiomeanalyst.ca/
