# Supplementary material for: Comparative Microbiota Composition Across Developmental Stages of Natural and Laboratory-Reared Chironomus circumdatus Populations From India
Source: Front Microbiol. 2021 Nov 26;12:746830. doi: 10.3389/fmicb.2021.746830 (PMC8661057; doi:10.3389/fmicb.2021.746830)
Supplement: Supplementary file 1 [file Data_Sheet_1.pdf]

Supplementary Material

Supplementary Figures and Tables

Supplementary Tables

Supplementary Table 1. A list of *C. circumdatus* samples labeled by sample location and *Chironomus* life stage. Samples were randomly collected from the three sampling locations, and the chironomid species was assigned only after DNA was extracted from each sample. All the samples that were identified as belonging to another *Chironomus* species (not shown) were excluded from the current study.

	**Egg mass (n)**	**Larva (n)**	**Pupa (n)**
**Mula River**	12	19	10
**Mutha River**	7	4	5
**Laboratory culture**	7	32	6

Supplementary Table 2. (Presented in an Excel file) **Sequencing coverage per sample specifying the number of reads that remained following each of the filtration steps during the bioinformatic analysis**.
Input: number of paired-end sequences after Illumina sequencing. Non-chimeric: after removing chimeric sequences. Filter1_ reads: number of reads after removing ASVs of non-bacterial origin (Archaea, chloroplast, mitochondria; unclassified at phylum level) and sequences with sequence lengths below 260 bp or above 262 bp. Filter1_ASVs: number of ASVs after filter 1. Filter2_reads: number of reads after removing ASVs with <50 reads across the entire dataset. Filter2_ASVs: number of ASVs after filter 2.
EP1, egg mass from Mula River; LBP1, LCP1, LDP1, larva from Mula River; PP1, pupa form Mula River; EP2, egg mass from Mutha River; LDP2, LCP2 Larva from Mutha River; PP2, pupa form Mutha River; EBL, egg mass from laboratory culture; LBBL, LCBL, LDBL larva from laboratory culture; PBL, pupa from laboratory culture.

Supplementary Table 3. (Presented in an Excel file) **ASV abundances within each sample.**
Specimens of three different life stages - egg masses, larvae, pupae were sampled from three sampling locations - Mula and Mutha Rivers and the laboratory culture, India. The table includes the ASVs before filter 2 (Supplementary Table 2), and taxonomic identification were applied.
EP1, egg mass from Mula River; LBP1, LCP1, LDP1, larva from Mula River; PP1, pupa form Mula River; EP2, egg mass from Mutha River; LDP2, LCP2 Larva from Mutha River; PP2, pupa form Mutha River; EBL, egg mass from laboratory culture; LBBL, LCBL, LDBL larva from laboratory culture; PBL, pupa from laboratory culture.

Supplementary Table 4. (Presented in an Excel file) **ASV taxonomic classification and abundances within each sample (life stages - egg masses, larvae, pupae, and sampling locations - Mula and Mutha Rivers and the laboratory culture, India).**
The Table includes ASVs with the abundances above 50 per ASV.
EP1, egg mass from Mula River; LBP1, LCP1, LDP1, larva from Mula River; PP1, pupa form Mula River; EP2, egg mass from Mutha River; LDP2, LCP2 Larva from Mutha River; PP2, pupa form Mutha River; EBL, egg mass from laboratory culture; LBBL, LCBL, LDBL larva from laboratory culture; PBL, pupa from laboratory culture.

Supplementary Table 5. Average ASV relative abundances at the phylum level. All the life stages at each sampling point are presented. *Proteobacteria* was the most dominant phylum in the environmental and laboratory-reared egg masses and pupal samples and in the laboratory larvae. *Fusobacteria* was the most dominant phylum in the environmental larvae. The results are presented as mean ± standard error of the mean (SEM). Phyla with abundances over 10.0% are marked in bold. *, unidentified (n=102).

**Phylum**	**Mula River**			**Mutha River**			**Laboratory culture**		
	**Egg mass**	**Larva**	**Pupa**	**Egg mass**	**Larva**	**Pupa**	**Egg mass**	**Larva**	**Pupa**
Actinobacteria	1.16±0.29	0.15±0.08	0.19±0.05	1.61±0.09	1.28±0.64	0.03±0.03	0.28±0.20	1.34±0.25	0.08±0.03
Bacteroidetes	**13.81±1.54**	**14.10±1.71**	**14.97±4.56**	**19.93±1.61**	8.53±0.91	4.69±0.97	**23.31±1.46**	**27.71±2.00**	**14.69±2.29**
Cyanobacteria	8.30±3.44	0.66±0.26	2.85±1.48	2.66±0.81	0.77±0.66	0.08±0.03	9.46±2.01	0.07±0.02	0.38±0.32
Epsilonbacteraeota	4.49±0.79	3.21±0.72	6.45±2.87	3.18±0.48	3.79±2.12	**16.40±2.97**	2.23±0.27	0.39±0.09	5.16±3.28
Firmicutes	2.40±0.39	**17.00±3.12**	7.63±2.21	3.53±0.51	4.87±1.85	2.33±1.26	6.31±1.77	**15.42±1.58**	5.58±3.40
Fusobacteria	0.23±0.04	**42.94±4.12**	1.54± 0.88	0.09±0.03	**47.70±10.28**	0.01±0.00	3.45±1.15	**13.52±2.26**	0.13±0.09
Proteobacteria	**67.90±3.78**	**12.47±3.91**	**63.34±3.91**	**64.02±2.21**	**30.59±8.59**	**74.51±2.91**	**53.22±3.04**	**37.51±2.75**	**71.48±4.36**
RsaHf231	0.00±0.00	0.95±0.32	0.02±0.02	0.00±0.00	0.44±0.22	0.00±0.00	0.10±0.09	0.70±0.13	0.00±0.00
NA*	0.73±0.14	8.15±1.34	1.96±0.85	2.93±0.32	1.79±0.76	1.47±1.04	0.58±0.15	1.49±0.24	1.75±0.68
Other	0.99±0.14	0.36±0.12	1.04±0.24	2.05±0.41	0.24±0.10	0.47±1.14	1.06±0.13	1.85±0.24	0.76±0.32

Supplementary Table 6. Statistically differentially abundant ASVs following linear discriminant analysis Effect Size (LEfSe). False discovery rate correction (FDR) and LDA scores are presented for the ten bacterial ASVs that contributed the most to differences in microbiota composition between the three sampled locations. See also supplementary Figure 2.

	LDA score	P-value	FDR
Burkholderiaceae (ASV 04)	5.79	1.06E-10	3.49E-09
C39 (*Rhodocyclaceae,* ASV 14)	5.69	1.16E-05	8.53E-05
Vibrio (ASV 07)	5.65	1.18E-06	1.33E-05
Burkholderiaceae (ASV 37)	5.46	5.65E-07	7.33E-06
Arcobacter (ASV 21)	5.35	6.02E-04	2.16E-03
Sphaerotilus (ASV 22)	5.31	4.61E-02	6.52E-02
Bacteroidia (ASV 12)	5.24	2.42E-06	2.41E-05
Flavobacterium (ASV 29)	5.22	4.33E-03	9.35E-03
Gottschalkia (ASV 10)	5.19	3.54E-03	8.08E-03
Bacteroidia (ASV 28)	5.14	2.60E-10	1.51E-08

Supplementary Figures

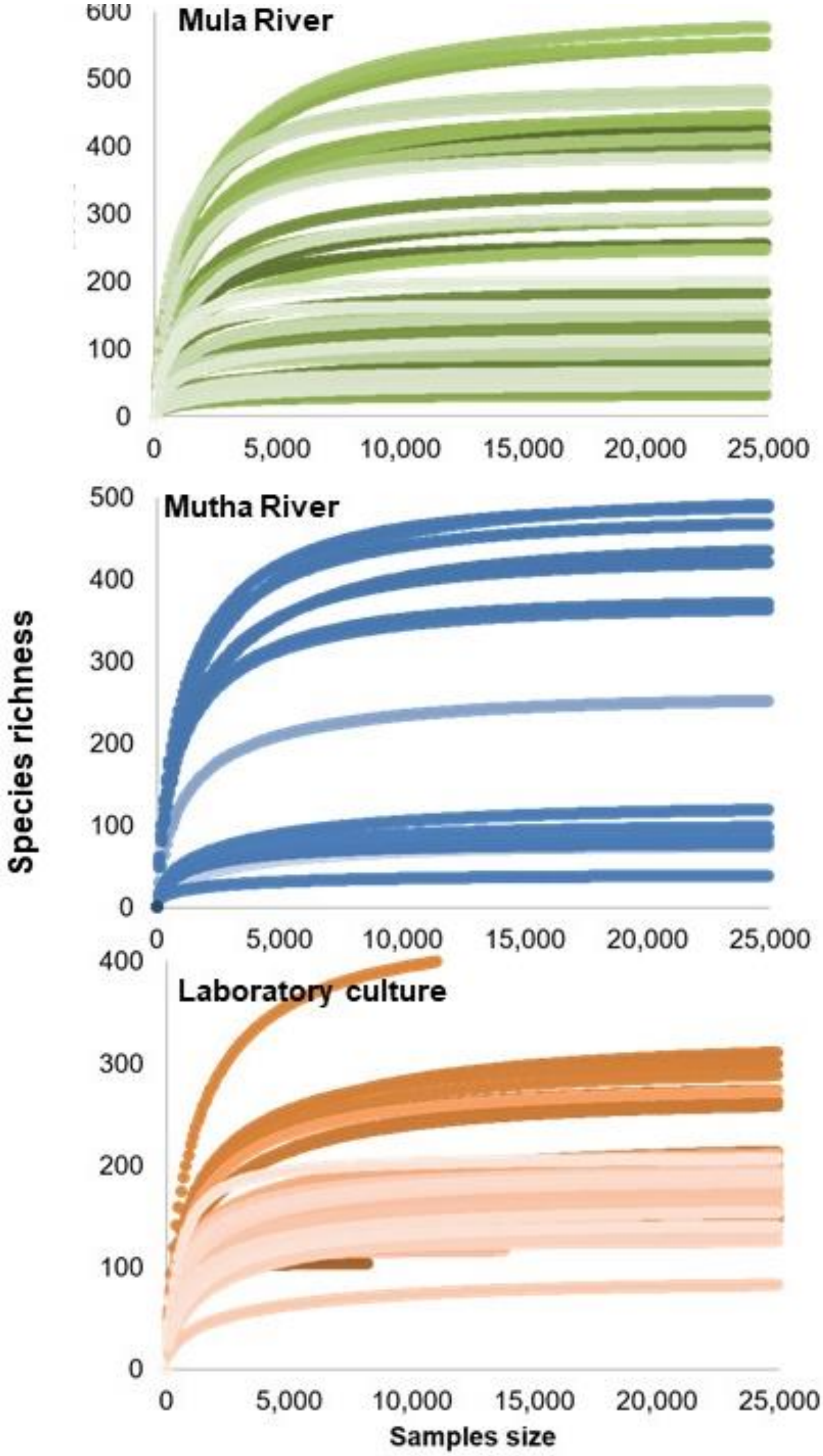

Supplementary Figure 1. **Rarefaction curves indicating the observed number of Amplicon Sequence Variants (ASVs) in all chironomid samples.** The rarefaction curves represent the increase in the number of ASVs as a function of the sequences number of each individual sample. The saturated shapes of the rarefaction curves indicate that the present sequencing depth recovered all of the bacterial species in each sample. The figures represent the microbiota of the three life stages that were sampled from the three different sampling locations.

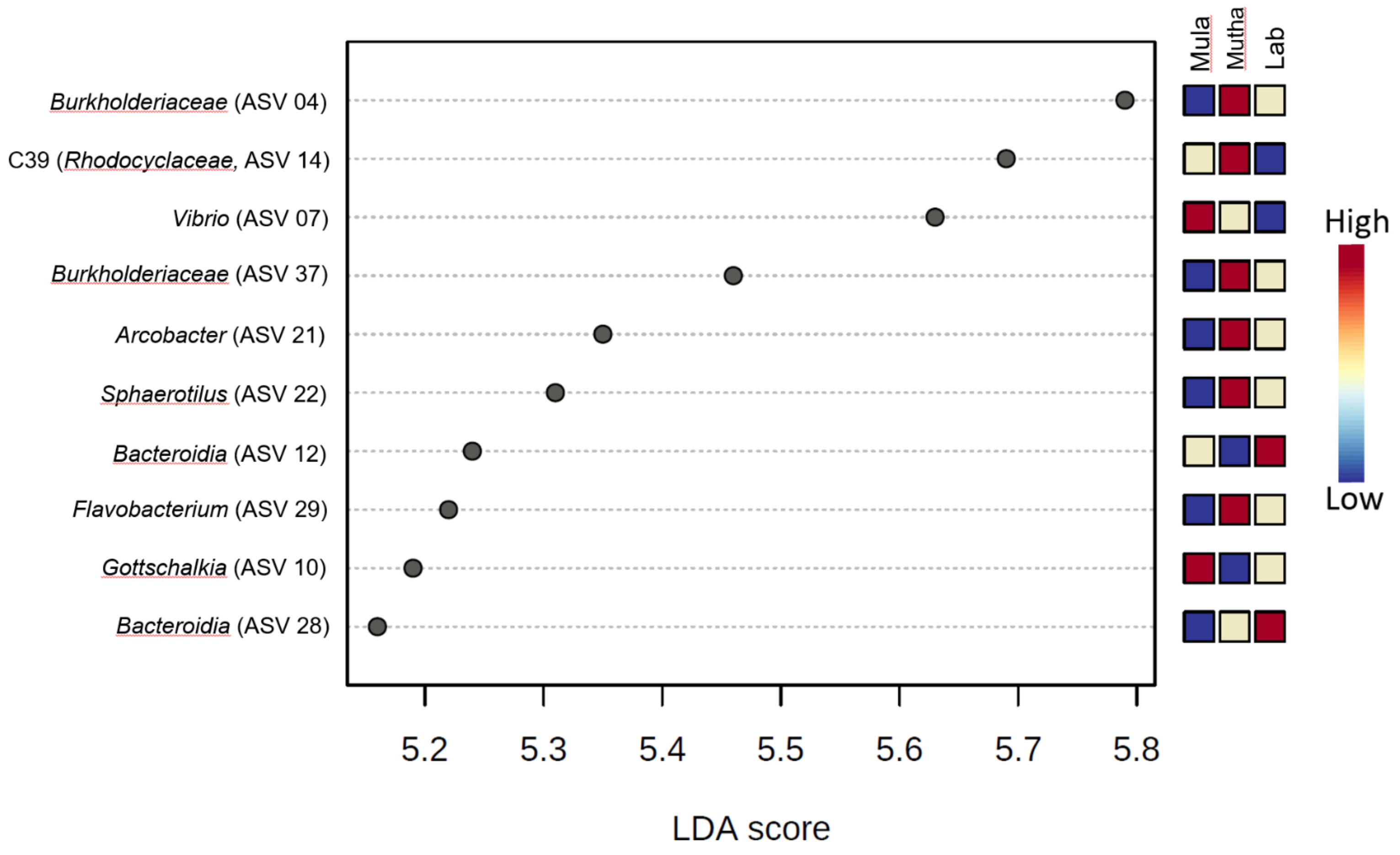

Supplementary Figure 2. **Linear discriminant analysis Effect Size (LEfSe) scores** displaying the ten bacterial ASVs that contributed the most to differences in microbiota composition between the three sampled locations. Colors indicate variations of bacterial abundances at each location, according to the color bar at the right. Statistical significance is presented in Supplementary Table 6.
